# m6A methylation: a new frontier in epilepsy research and therapeutics

**DOI:** 10.17179/2025-8359

**Published:** 2025-05-30

**Authors:** Mudasir Maqbool, Yumna Khan, Mohammed M. Arab, Saud O. Alshammari, Md Sadique Hussain, Fawaz M. Almufarriji

**Affiliations:** 1 Department of Pharmacology, Government Medical College, Baramulla, Jammu and Kashmir 193103, India; 2 Institute of Biotechnology and Genetic Engineering (Health Division), The University of Agriculture, Peshawar 25000, Khyber Pakhtunkhwa, Pakistan; 3 Department of Biochemistry, Faculty of Science, University of Tabuk, Tabuk, 71421 Saudi Arabia; 4 Department of Pharmacognosy and Alternative Medicine, College of Pharmacy, Northern Border University, Rafha, Saudi Arabia; 5 Uttaranchal Institute of Pharmaceutical Sciences, Uttaranchal University, Prem Nagar, Dehradun 248007, Uttarakhand, India; 6 Department of Clinical Laboratory Sciences, College of Applied Medical Sciences, Al- Quwayiyah, Shaqra University, Riyadh, Saudi Arabia

**Keywords:** epilepsy, m6A modification, m6A regulators, neurodegeneration, precision medicine, molecular biomarkers

## Abstract

Epilepsy is a highly complex and global neurological disorder, for which available treatments only inadequately control the disease in many patients. Recent advances in molecular research have identified N6-methyladenosine (m6A) RNA modifications as key regulators of neuronal processes that underpin the pathophysiology of epilepsy. This review critically discusses the emerging significance of m6A modifications in epilepsy, focusing on dynamic regulations of m6A "writers," "erasers," and "readers" for modulating gene expression, neuronal excitability, and synaptic plasticity in epilepsy. Dysregulation of m6A machinery promotes epilepsy by exacerbating oxidative stress, mitochondrial dysfunction, and neuronal damage. We also discuss the prognostic significance of m6A alterations as a potential biomarker in epilepsy diagnosis and disease progression, along with advanced therapeutic strategies against m6A, including small molecules, RNA editing technologies, and precision medicine. This review highlights the transformational significance of m6A modulation in epilepsy therapy and opens new avenues for personalized therapeutic strategies that may revolutionize the field of drug-resistant epilepsy and improve the prognosis for patients.

See also the graphical abstract(Fig. 1).

## Introduction

**Figure 2:**
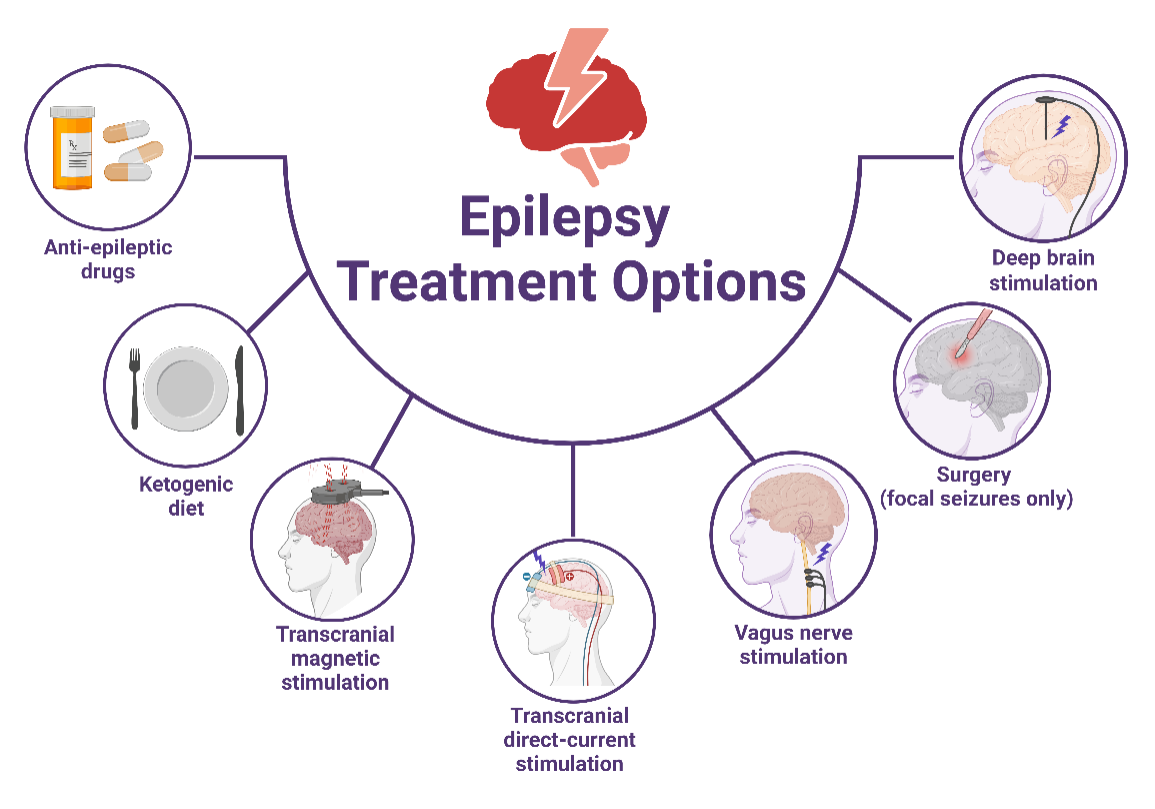
Current epilepsy treatment options

Epilepsy is among the most widespread neurological conditions worldwide (Engel, 2011[33]). Roughly 100 million individuals will experience at least one epileptic seizure in their lifetime, resulting in significant social, physical, economic, and psychological ramifications (Aguiar et al., 2012[1]; Diop et al., 2003[28]; Hussain et al., 2024[51]). The lifetime prevalence of epilepsy is estimated to be a median of 5.8 per 1,000 people in developed regions and 10.3 per 1,000 in developing areas (Aguiar et al., 2012[1]; Ngugi et al., 2010[95]). Epilepsy, a brain disorder, is characterized by certain conditions: either two unprovoked (or reflex) seizures occurring over 24 hours apart, a single unprovoked (or reflex) seizure with a high probability (at least 60 %) of recurrence within the next 10 years, or a confirmed diagnosis of an epilepsy syndrome (Fisher et al., 2014[37]; Ma et al., 2024[83]). Figure 2(Fig. 2) illustrates the available treatment options for epilepsy. According to the International League Against Epilepsy (ILAE), an epileptic seizure is a temporary occurrence marked by abnormal and excessive neuronal activity in the brain (Fisher et al., 2017[38]). The prolonged neuronal excitation seen during seizures triggers a cascade of cellular processes, such as the activation of glutamate receptors and cytokines, changes in the composition of glutamate and gamma-aminobutyric acid receptors, and modifications in neuronal plasticity (Chuang, 2010[21]). The role of mitochondrial dysfunction and oxidative stress (OS) in the development of neurological disorders, including epilepsy, is progressively gaining recognition (Patel, 2004[97]; Qian et al., 2023[104]). The relationship between OS and epilepsy is yet to be fully understood; it is uncertain whether OS triggers epilepsy or results from it. However, sustained neuronal excitation during seizures intensifies the generation of reactive oxygen species (ROS). This phenomenon arises when unpaired electrons leak from the electron transport chain and combine with molecular oxygen to form superoxide, thereby exacerbating seizure-induced brain injury (Méndez-Armenta et al., 2014[89]). 

Recent discoveries in epitranscriptomics have shown the dynamic role of N6-methyladenosine (m6A) methylation in controlling gene expression related to a various of physiological processes (Chen et al., 2024[16]). As the most prevalent internal modification in eukaryotic mRNAs, m6A regulates various critical steps of mRNA metabolism, including splicing, export, translation, and stability (Wang et al., 2022[138]). This is mediated by "writers" such as methyltransferase-like 3 (METTL3) , methyltransferase-like 14 (METTL14), and Wilms' tumor 1-associating protein (WTAP) that add methyl groups, while "erasers" include alkylation repair homolog protein 5 (ALKBH5) and Fat mass and obesity-associated protein (FTO) that remove them, and "readers" such as YTH domain-containing proteins recognize m6A sites to impact RNA fate (Zaccara et al., 2019[162]). The capability of m6A methylation to enable rapid adjustments in gene expression in reaction to environmental and physiological changes is especially important in the brain, where it prominently influences neurodevelopment and aging (Shafik et al., 2021[113]). Abnormal levels of m6A methylation are linked with aging-related changes in the central nervous system (CNS) and various neurological disorders, including multiple sclerosis Alzheimer's disease (AD), Parkinson's disease (PD), and notably epilepsy (Zhang et al., 2022[166]). 

Recent findings highlight the critical role of m6A methylation in epilepsy, mainly in seizure susceptibility and disease development. The glutamate-cystine antiporter (xCT), targeted by the m6A reader protein YTHDC2, has been recognized as a key factor in temporal lobe epilepsy (TLE) with hippocampal sclerosis. Suppressing xCT or YTHDC2 activity in reactive astrocytes reduces glutamate concentrations. This highlights the role of m6A in glutamate dysregulation and seizure pathophysiology (Zhang et al., 2024[164]). Additionally, m6A modifications target genes involved in epileptogenesis, including those responsible for neuronal excitability, inflammation, and synaptic function. High seizure frequency and neuronal apoptosis are associated with increased m6A methylation of nuclear factor erythroid 2-related factor 2 (Nrf2) mRNA, underlining its role in the pathogenesis of epilepsy (Tian et al., 2023[131]). Moreover, a research study demonstrated that m6A modification is highly involved in epilepsy through the regulation of gene expression involving a transcription factor, SRF, and METTL3. In SRF knockout models, the reduction of m6A modification over-expresses the GEM gene, contributing to neuronal damage, while SRF overexpression enhances the activity of METTL3, hence promoting m6A modifications to mitigate OS and inflammation within epileptic neurons (Li and Liu, 2024[68]).

This evolving understanding opens promising avenues, from novel diagnostic and prognostic approaches to new therapeutic options for the connection between m6A and epilepsy. Investigation into the role of m6A methylation in epilepsy may provide new biomarkers for diagnosis and outcome prediction and targeted therapies aimed at seizure management and prevention of disease progression. This work seeks to clarify the function of m6A methylation in epilepsy, providing a novel pathway for investigation and therapies that have the potential to greatly enhance our comprehension and treatment of this incapacitating condition.

## m6A RNA Modifications

**Figure 3:**
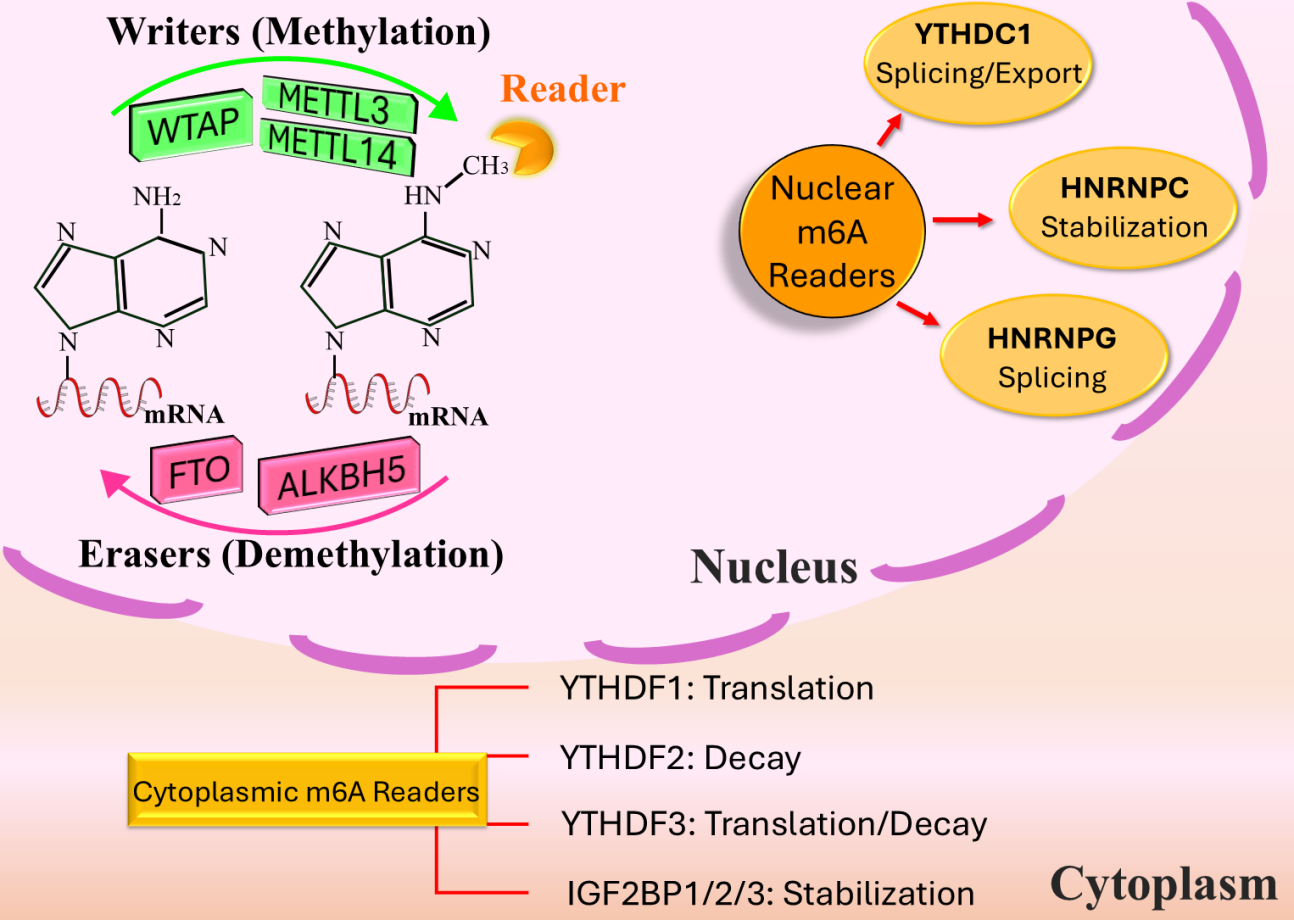
Overview of the mechanisms of modification of m6A, highlighting writers, erasers, and readers along with their roles in RNA regulation

The curiosity about the m6A alteration has significantly increased in the past few years with the identification of enzymes responsible for adding, removing, and recognizing this change. The association between m6A and mRNA has been uncovered. The m6A alteration is present in the majority of transcripts, and the m6A/A ratio in mRNAs varies from 0.2 % to 0.5 % (Li et al., 2020[69]). Physiological activities like mRNA nuclear export, translation, and destruction include reversible and dynamic m6A changes. These alterations are strongly associated with cancerous cell growth, death, spread, and susceptibility to treatment. m6A is also abundant in non-coding RNAs (ncRNAs) (Guo et al., 2024[41]). For instance, it facilitates tumor progression in lung cancer by increasing the levels of LCAT3, a long ncRNA (lncRNA) (Qian et al., 2021[106]). Figure 3(Fig. 3) summarizes the molecular mechanisms underlying the m6A modification by depicting important players including writers (methyltransferases), erasers (demethylases), and readers, and their functions in regulating various RNA processes.

### Writers

The identification of a methyltransferase complex (MTC) that modifies newly synthesized pre-mRNAs by m6A methylation has significantly increased the level of significance in m6A. Writers refer to a collection of m6A MTC (MAC) that facilitate the attachment of methyl groups to RNA (Sun et al., 2021[122]; Wang et al., 2017[140]). The m6A modification of mRNA is facilitated by the MAC, which comprises METTL3, METTL14, and the m6A-METTL-associated complex (MACOM). MACOM consists of five proteins: Zinc finger CCCH-type containing 13 (ZC3H13), WTAP, Vir-like m6A methyltransferase-associated (VIRMA/KIAA1429), RNA binding motifs protein 15 (RBM15), and Cbl proto-oncogene like 1 (HAKAI/CBLL1) (Lence et al., 2019[62]). In vitro, the heterodimeric combination of METTL3 and METTL14, along with ZC3H13, is the minimum need for exerting m6A alterations. The N6-methylation process, facilitated by METTL3, utilizes S-adenosylmethionine (SAM) as the methyl contributor. METTL14 performs an essential part in enhancing METTL3 function by attaching to the substrate RNA and arranging the methyl group for transport to adenosine (Wang et al., 2016[136]). The majority of m6A domains in the METTL3/METTL14 complex are concentrated in the 3' untranslated regions (UTRs) and near-stop codons (Dominissini et al., 2012[29]; Meyer et al., 2012[92]). WTAP interfaces with the METTL3-METTL14 complex, enabling it to be restricted to nuclear speckles alongside pre-mRNA processing molecules. It functions to control MAC recruiting to mRNA targets for the catalytic process in vivo. When WTAP is not present, the ability of METTL3 to attach to RNA is significantly diminished (Ping et al., 2014[102]). 

The fundamental complex comprises METTL3, METTL14, and WTAP, while the linker proteins KIAA1429, RBM15, HAKAI, and ZC3H13 may interact with heteropolymers and collaborate with the primary MTC to establish the accurate localization of MAC. Human KIAA1429 is the most significant element in the MAC. It consists of a C-terminal (C-KIAA1429) and N-terminal (N-KIAA1429) and mostly facilitates mRNA methylation in the 3'UTR and close to the stop codon (Cui et al., 2022[24]; Yue et al., 2018[161]). KIAA1429 perhaps acts as a framework connecting the catalytic core components METTL3/METTL14/WTAP and RNA substrates, influencing the targeted placement of m6A via its N-KIAA1429 domain (Qian et al., 2019[105]). ZC3H13 serves as an intermediary, linking the RNA-binding protein RBM15 to WTAP (Knuckles et al., 2018[56]). AKAI, also known as CBLL1, is a preserved component of the MACOM. Its ubiquitination region plays a crucial role in preserving the stability of the MACOM. HAKAI, in line with its function in the m6A route, additionally serves a part in the sex-determined system and facilitates the splicing of sex-related factors (Bawankar et al., 2021[7]; Wang et al., 2021[143]).

Investigation into the organization and operation of human m6A writers is thriving. Recent research has discovered that METTL16, an additional enzyme, is a newly found m6A methyltransferase (METT). METTL16 has an RNA-binding domain at its N-terminus and functions as a METT. It can communicate with several types of RNAs, such as MAT2A mRNA, MALAT1 lncRNA, and U6 snRNA (Pendleton et al., 2017[98]; Satterwhite and Mansfield, 2022[112]; Warda et al., 2017[144]). Additionally, it employs SAM as a methyl donor, much as METTL3 (Doxtader et al., 2018[32]; Satterwhite and Mansfield, 2022[112]). The RRACH domain of METTL16 differs from that of METTL3/METTL14 in that it demands both a UACAGAGAA consensus motif and a specific stem-loop RNA framework. METTL16 influences many m6A changes in the epidermal transcriptome and regulates the splicing of SAM synthase genes to maintain SAM equilibrium (Ruszkowska, 2021[111]). The METTL5/TRMT112 complex has been lately shown to function as a METT that is associated with the alteration of 18S rRNA at the m6A site. Additionally, ZCCHC4 is an enzyme that plays a significant role in the alteration of 28S rRNA at the A4220 site (Pinto et al., 2020[103]; van Tran et al., 2019[132]). Like METTL3/METTL14, METTL5 functions as the catalytic component of the complex. Additionally, TRMT112 potentially serves in RNA binding and activating METTL5, hence enhancing its relationship with SAM. The two proteins are connected by a parallel β-zipper, and METTL5 is stabilized by TRMT112 by the covering of a significant hydrophobic region on it (van Tran et al., 2019[132]). PCIF1, a cap-specific adenosine METT, has been demonstrated to modify the m6A of 2'-O-methyladenosine (Am) in eukaryotic mRNA. This modification results in the formation of the m7Gpppm6 Am arrangement, but only when Am serves as the initial nucleotide transcribed (Akichika et al., 2019[2]; Sun et al., 2019[121]).

### Erasers

Erasers refer to a collection of proteins that eliminate the methyl groups from RNA fragments that have been mutated with m6A. This includes two specific kinds of demethylating enzymes, namely FTO and ALKBH5. The introduction of erasers renders m6A capable of undergoing dynamic and reversible changes. FTO was the primary m6A demethylase to be discovered and it is found in nuclear speckles and the cytoplasm. Research has shown that FTO has proficient oxidative demethylation ability towards several m6A sites in RNA (Jia et al., 2011[52]). FTO enzyme may also target N6, 2'-O-dimethyladenosine (m6Am) in mRNA and snRNA, along with m6A. This interaction is important for mRNA stability as it helps resist DCP2-mediated mRNA decapping, as shown in studies (Mauer et al., 2017[87]; Wei et al., 2018[146]). FTO enzyme facilitates the removal of the methyl group from m6A specifically in the nucleus, although it can regulate the removal of methyl groups from both m6A and m6Am in the cytoplasm. There is disagreement on the preference of FTO for m6A and m6Am, although it is generally agreed that FTO has a greater preference for m6A in the nucleus and a stronger preference for m6Am in the cytoplasm (Jia et al., 2011[52]; Mauer et al., 2017[87]). 

ALKBH5 is a well-known protein that can remove m6A methylation in mammals. It has a wide-ranging impact on nuclear RNA metabolism, export, and gene expression, and is involved in essential procedures via its demethylation action both in vivo and in vitro. ALKBH5 removes the methyl group from single-stranded RNA molecules containing m6A, and its action is like that of FTO (Zheng et al., 2013[175]). FTO and ALKBH5 are members of the iron and 2-oxoglutarate-dependent family of AlkB oxygenases, although they have different physiological activities. FTO is strongly associated with obesity, whereas ALKBH5 plays a crucial role in spermatogenesis (Church et al., 2010[22]; Zheng et al., 2013[175]). FTO has high expression levels in the brains of mice, while ALKBH5 shows high expression levels in the testes. FTO and ALKBH5 may selectively catalyze the demethylation of certain target mRNAs to produce distinct biological effects.

### Readers

Proteins that specifically attach to the methylation site of m6A are referred to be readers or m6A recognition proteins. They specifically identify m6A modifications on targeted RNA and participate in many RNA metabolic pathways. Readers comprise YTHDC1-2 and YTHDF1-3 (YTH domain-containing proteins), IGF2BP1-3 (insulin-like growth factor 2 mRNA-binding proteins), and hnRNPA2B1 and HNRNPC/G (heterogeneous nuclear ribonucleoproteins). 

YTHDC1 is situated inside the nucleus, whereas YTHDC2 and YTHDF1-3 are situated within the cytoplasm (Hsu et al., 2017[46]; Shi et al., 2017[115]; Xiao et al., 2016[152]). YTHDC1 interacts with and brings in KDM3B, an enzyme that removes a specific chemical modification (dimethylation) from a histone protein called H3 at a specific location (lysine 9). This interaction occurs in areas of chromatin that are connected with a chemical modification called m6A. The removal of the dimethylation from H3K9me2 by KDM3B, facilitated by YTHDC1, results in enhancement of gene expression (Li et al., 2020[70]). YTHDC1 regulates the process of splicing, nuclear-cytoplasmic export, and destruction of RNAs that have been altered with m6A by controlling the activity of splicing variables and the selection of the nuclear exosome for nuclear destruction (Liu et al., 2020[73]; Roundtree et al., 2017[110]; Xiao et al., 2016[152]). On the other hand, YTHDC2 reduces the viability of mRNA that has been altered with m6A by engaging with RNA helicase. However, it enhances the effectiveness of translating specific mRNA (Mao et al., 2019[86]; Tanabe et al., 2016[124]; Wojtas et al., 2017[148]). 

While YTHDF have a comparable structure, their activities vary. YTHDF1, YTHDF2, and YTHDF3 interact with certain mRNAs and regulate the stability and translation of the mRNAs associated with YTHDF proteins (Lan et al., 2021[60]; Ries et al., 2019[109]; Wang et al., 2014[141]). YTHDF1 enhances the process of translation and improves the accuracy of translating m6A-tagged transcripts, resulting in increased protein synthesis (Wang et al., 2015[142]). The YTHDF2-mRNA complex consists of two structural domains: the C-terminal domain binds m6A-mRNA, while the N-terminal domain is important for localizing the complex to cellular RNA degradation domains (Wang et al., 2014[141]). YTHDF3 promotes protein synthesis via YTHDF1 and enhances the breakdown of methylated mRNA through YTHDF2 (Shi et al., 2017[115]). The three YTHDF proteins have a broad and coordinated impact on essential biological processes related to m6A RNA methylation. 

IGF2BPs enhance the stabilization of RNA and augment mRNA retention under fluctuating physiological circumstances by enlisting RNA stabilizers including matrin 3. RNA stabilizers consist of ELAV-like RNA-binding protein 1 and poly (A) -binding protein cytoplasmic 1 (Huang et al., 2018[48]; Lin et al., 2016[72]). 

Heterogeneous nuclear ribonucleoproteins (hnRNPs) have many functions in the control of transcriptional and posttranscriptional expression procedures such as RNA splicing, alterations, translation, and destruction (Liu et al., 2024[78]; Wu et al., 2018[149]). Alarcon et al. provided evidence that hnRNPA2B1 functions as a nucleic acid reader for m6A modification and enhances the processing of a specific group of pri-miRNAs that are reliant on METTL3. hnRNPA2B1 can attach to nuclear RNAs which include the G (m6A) C sequence both in living organisms and in laboratory settings. It can also bring together the microprocessor Drosha-DGCR8 complex and safeguard the RNA target regions from being broken down by ribonucleases (Alarcón et al., 2015[3]). Li et al. demonstrated that the m6A methylation of LINC01 833, induced by METTL3, enhances the advancement of non-small cell lung cancer via controlling hnRNPA2B1 (Li et al., 2022[64]). According to Wu et al. (2018[149]), m6A may boost the capacity of hnRNPA2B1 to promote nuclear processes, including pri-miRNA processing. This is achieved via enhancing the reach of hnRNPA2B1 to specific binding regions, rather than attachment to m6A. Further investigation is required to unravel the complexities of the m6A mechanism.

### m6A modifications as epigeneticregulators of gene expression

Unlike DNA methylation or histone modifications, which operate at the chromatin level, m6A directly affects the fate of RNA transcripts post-transcriptionally, thereby controlling splicing, nuclear export, translation efficiency, and degradation. This positions m6A as a distinct yet powerful epigenetic mark within the broader epitranscriptomic landscape (Cao et al., 2024[13]; He and He, 2021[44]). What makes m6A modifications truly epigenetic is their responsiveness to environmental and developmental cues, enabling them to modulate gene expression without changing the underlying nucleotide sequence. For instance, in neural stem cells, m6A-mediated regulation of RNA stability affects lineage-specific gene programs critical for differentiation (Dong et al., 2024[30]; Zhang et al., 2022[166]). In embryonic development and stem cell renewal, the methylation landscape dynamically shifts in response to extrinsic signals, underscoring m⁶A's pivotal role in developmental plasticity (Wen et al., 2023[147]).

### Crosstalk between m6A modifications and extrinsic signaling pathways

**Figure 4:**
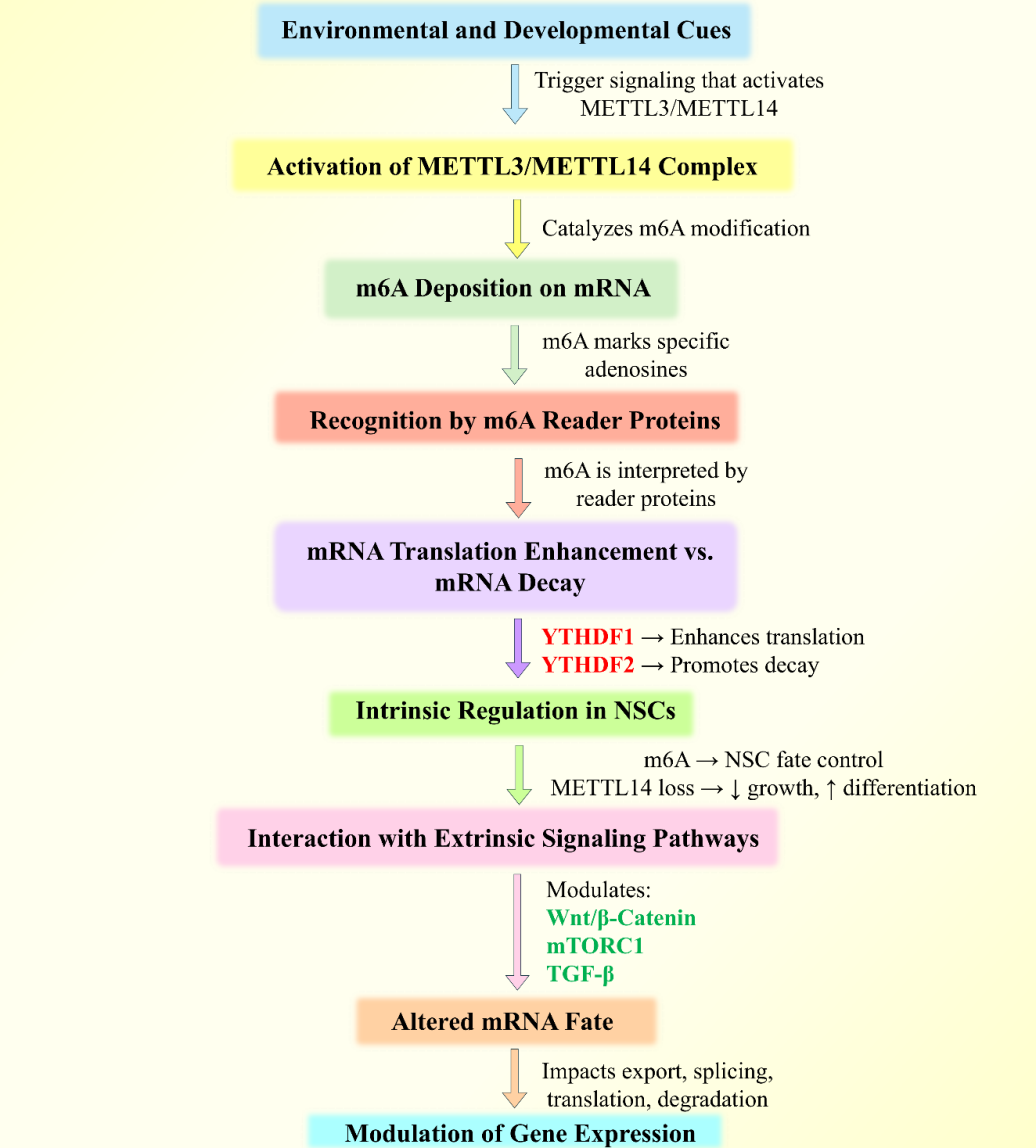
Schematic representation of the integration between extrinsic signaling pathways (e.g., NF-κB, mTORC1, and Wnt/β-catenin) and the m6A RNA modification machinery. External cues such as stress, cytokines, and nutrients activate intracellular pathways that regulate the expression or activity of m6A writers, erasers, and readers. These modifications influence RNA fate, stability, degradation, or translation, ultimately affecting gene expression involved in inflammation, regeneration, and tumorigenesis.

Recent research has uncovered that m⁶A does not act in isolation but is intimately connected with numerous extrinsic signaling pathways that control cellular responses to the environment (Figure 4(Fig. 4)). One prominent example is the NF-κB pathway, a key regulator of inflammation. METTL14-dependent m⁶A methylation has been shown to modulate the stability of NF-κB target mRNAs such as IL-6, thereby influencing macrophage activation and inflammatory states in atherosclerosis (Zheng et al., 2022[176]). In another study, mTORC1 signaling, which responds to nutrient availability and cellular stress, was found to elevate S-adenosylmethionine (SAM) levels-the primary methyl donor for m⁶A methylation-subsequently enhancing m6A deposition and stress-responsive gene expression (Cheng et al., 2023[19]).

The Wnt/β-catenin pathway, central to cell proliferation and embryogenesis, also appears to be regulated by m⁶A modifications. Wu et al. (2024[151]) demonstrated that METTL3 upregulates Wnt target genes by promoting their m⁶A-mediated translation, revealing a feedback loop that sustains oncogenic signaling. Similarly, Hedgehog signaling, which governs tissue patterning and regeneration, is modulated through m⁶A-dependent transcript decay, fine-tuning the pathway's activity during liver fibrosis and neural development (Huang et al., 2024[47]; Wei et al., 2022[145]). In the TGF-β and Hippo pathways, m6A modifications act as gatekeepers of signal transduction fidelity by ensuring timely turnover of transcripts encoding key pathway components and transcription factors (Peng et al., 2025[100]). These observations converge on a unifying theme: m6A acts as a critical post-transcriptional node through which extrinsic signals sculpt cellular identity and behavior.

### Physiological roles of m6A modification in neuronal function

m6A is one of the major RNA modifications regulating a wide range of cellular events, including cell cycle, cell differentiation, and stress responses, and has especially been studied in neurons. m6A controls neurodevelopment by regulating the neuronal transcriptome with major impacts on neuronal functions (Livneh et al., 2020[79]). Recent studies have evidenced that m6A methylation regulates neuronal autophagy, an important cellular function that controls the pathways of cell survival and stress responses. High levels of m6A might interfere with autophagy, underscoring the dynamic function of m6A in RNA metabolism and its importance in maintaining neuronal health and resilience to stress (An et al., 2024[4]). 

m6A also performs a significant role in regulating certain mRNAs in neurons necessary to maintain neuronal activities and overall brain health (Tegowski et al., 2024[130]). Moreover, synaptic genes are controlled by the m6A modification, and it is shown that m6A methylation normally undergoes an age-related shift. For example, the synaptic genes that are hypermethylated in young mice became hypomethylated in older animals. Recently, three critical m6A regulatory enzymes, METTL3, YTHDF1, and FTO, have been identified as key modulators of synaptic function, and thus dysregulation of m6A may lead to age-related neurodegenerative processes (Chopra et al., 2024[20]). Furthermore, m6A modification has been reported to modulate synaptic plasticity and neuronal response through the regulation of the stability and translatability of mRNAs necessary for synaptic function by altered levels of m6A, thereby establishing its critical role in normal neuronal activity and implying a potential involvement in neurodegenerative diseases (Qiu et al., 2024[108]). 

**Figure 5:**
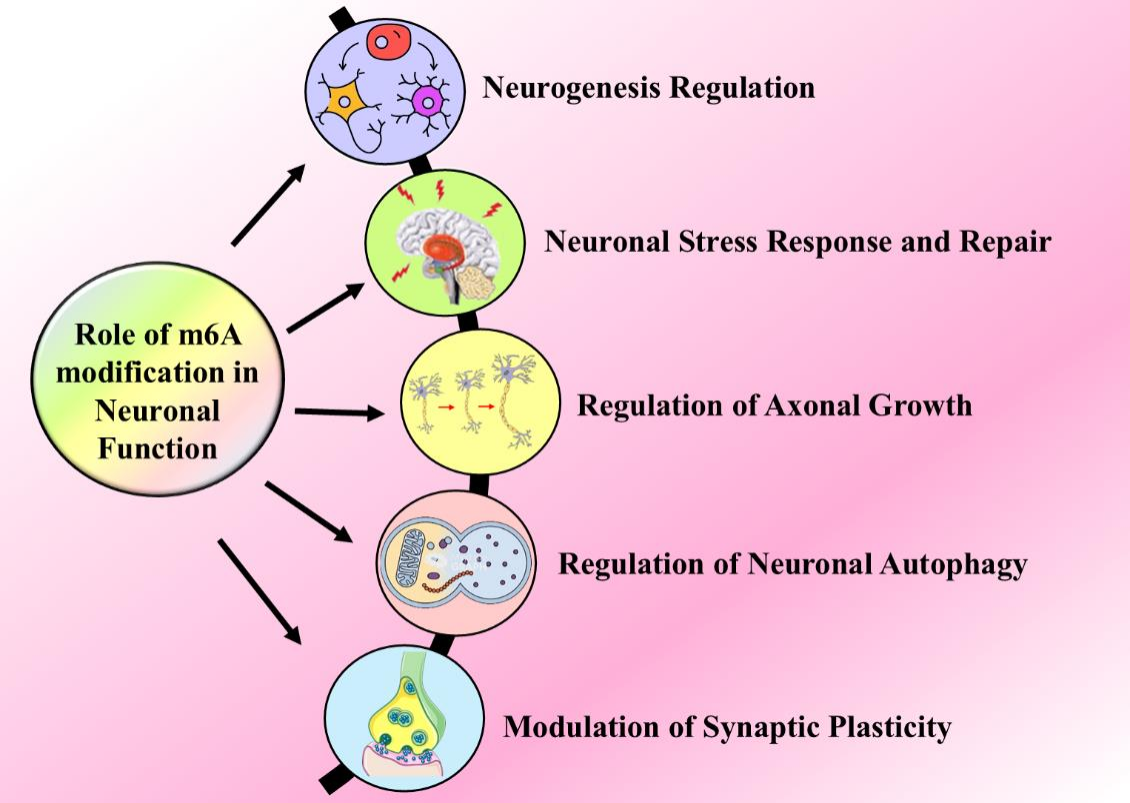
Physiological roles of m6A modification in neuronal function

Besides synaptic regulation, another essential participation of m6A modification is in neuronal stress responses and injury repair. One study demonstrated that exposure to bisphenol A (BPA) elevated METTL3 level and expression, which, in turn, enhanced the m6A level in hippocampal neurons, leading to cognitive impairments. Notably, inhibition of METTL3 reversed these effects and restored synaptic function, pointing toward a role for m6A in neuronal function with potential contributions to neurotoxicity (Li et al., 2023[65]). Furthermore, m6A modification controls axonal outgrowth and neuronal function in cerebellar granule cells. m6A reader proteins, including YTHDF1 and YTHDF2, modulate axon growth by interacting with key components of the Wnt signaling pathway, such as Dvl1 and Wnt5a, which are critical for the development of cerebellar parallel fibers and synapse formation (Yu et al., 2021[159]). In the context of ischemic injury, the modification of m6A is considered to regulate neural stem cell (NSC) behavior following ischemia/reperfusion. One study reported that elevated levels of m6A and METTL14 in NSCs after hypoxia/reoxygenation significantly influenced the proliferation, migration, and differentiation of NSCs. More importantly, the knockdown of METTL14 suppressed these regeneration processes, highlighting that m6A is critical for NSC activation and neuronal repair following ischemic injury (Zhang et al., 2024[168]). Additionally, the methylation of m6A, mediated by METTL3, facilitates cellular responses including apoptosis, inflammation, and autophagy in ischemia/reperfusion injury. m6A also regulates the expression of certain lncRNAs, including CRNDE, influencing mRNA stability and neuron survival, thereby further emphasizing the role of m6A in the injury and repair mechanisms of neurons (Yu et al., 2024[160]). Also, m6A modification of the lncRNA MEG3 has been demonstrated to modulate mitochondrial dysfunction and OS under ischemic injury conditions. In the animal models of ischemia, MEG3 overexpression, and its m6A modification exacerbated mitochondrial fragmentation and OS by enhancing apoptosis. Thus, this pathway presents a promising target for therapeutic strategies in ischemic stroke (Yao et al., 2024[156]). Collectively, these studies epitomize the multifaceted role of m6A in neuronal function, from the regulation of synaptic plasticity to the promotion of neuronal repair mechanisms, hence positioning the m6A modification as a potential pharmacological target for neurodegenerative diseases and ischemic conditions. Figure 5(Fig. 5) summarizes the physiological role of m6A modification in neuronal function and mainly focuses on how m6A modification influences different cellular processes in neurons.

## Dysregulation of m6A in Epileptic Pathophysiology

The dysregulation of m6A RNA modifications is considered an integral part of epilepsy pathophysiology, affecting neuronal survival, synaptic plasticity, and the overall development of the disease. m6A modification is regarded as one of the important factors in gene expression regulation. Dysregulation of this process in epilepsy has been related to several key processes, including autophagy, OS, and mitochondrial dysfunction. For example, m6A promotes the expression of TRPML1, a protein involved in autophagy and induced by seizure activity that helps cells bear the stress. On the other hand, when the function of the m6A regulation on TRPML1 is disrupted, the neuronal injury aggravates, pointing to a therapeutic potential of m6A toward neurotoxicity reduction in epilepsy (Peng et al., 2024[101]). Alterations in m6A modification have also been related to changes in the expression of key m6A regulators, such as HNRNPC, WTAP, RBM15, and YTHDC1, that impact disease susceptibility and progression. These factors further contribute to immune responses and metabolic changes in epileptic conditions, with predictive models identifying distinct m6A patterns that could serve as biomarkers for epilepsy (Liu et al., 2022[77]). The dysregulation of m6A further extends its impact on synaptic function, interacting with synaptic proteins such as piccolo and synapsin 1 and with myelin-related proteins, all important in maintaining neuronal integrity and communication. Thus, disruption in the modification of m6A, especially in synaptic proteins, indicates that such processes have significantly contributed to neuronal dysfunction and seizure pathophysiology (Aparicio et al., 2020[5]). Evidence from both animal models and human tissues has further underscored that dysregulated m6A methylation affects the neuronal structure and synaptic plasticity, important to maintain proper neuronal excitability. These mechanisms, including METTL3 inhibition, which affects m6A methylation, alter the structure of dendritic spines and postsynaptic density which are critical in synaptic plasticity (Benito et al., 2023[8]). Moreover, research on neonatal anesthesia exposure has documented that altered m6A methylation of critical brain regions results in long-lasting behavioral deficits and impaired synaptogenesis, which further implicates m6A dysregulation in epileptogenesis and neurodevelopmental disorders (Wu et al., 2023[151]). 

## Molecular Mechanisms of m6A in Epilepsy

**Figure 6:**
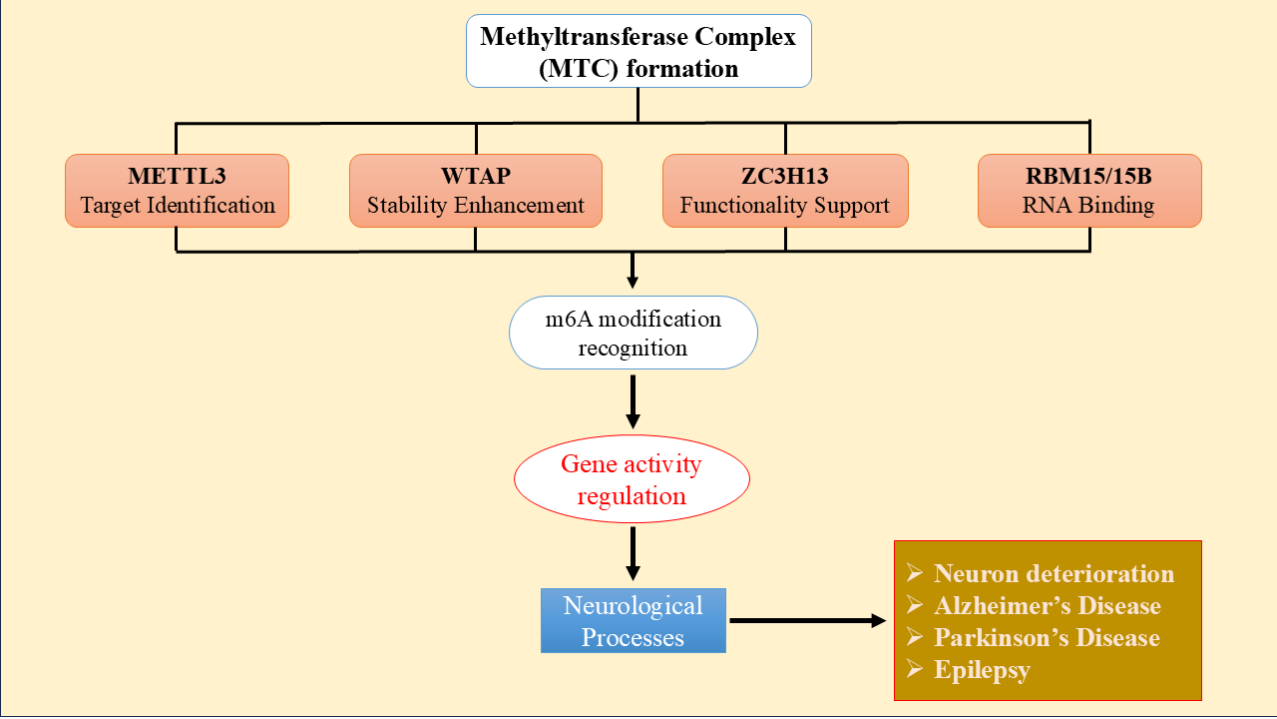
Role of the methyltransferase complex (MTC) in m6a modification and neurological disorders. This diagram illustrates the formation of the MTC and its key components, which contribute to m6A RNA modification through functions such as target identification, stability enhancement, functionality support, and RNA binding. The m6A modification subsequently influences gene activity regulation, impacting neurological processes that may lead to conditions like neuron deterioration, AD, PD, and epilepsy.

The primary emphasis of research in the field of epigenetic changes of eukaryotic mRNA has been on RNA methylation alterations, including m6A, 5-methylcytosine (m5C), N7-methylguanosine (m7G), and N1-methyladenosine (m1A) (Long et al., 2023[80]). Out of all the internal modifications of mRNAs and ncRNAs in organisms, m6A is widely regarded as the most prevalent (Tao et al., 2022[127]; Yang et al., 2022[155]). The m6A modification is regulated by a central MTC consisting of METTL3, METTL14, and WTAP (Figure 6(Fig. 6)) (You et al., 2022[157]). METTL14 facilitates the identification of targets by METTL3. WTAP interacts with the METTL3-METTL14 complex and enhances its stability, guaranteeing its precise localization in the nuclear region and initiating its enzymatic function. ZC3H13 primarily serves to sustain the functionality and arrangement of the MTC, which is situated inside the nucleus. The RBM15/15B protein may collaborate with the METTL3-WTAP intricate to facilitate the binding of MTC to the U-rich section of RNA (Tao et al., 2023[128]). Currently, two m6A demethylases have been discovered, namely FTO and ALKBH5 (Zhang et al., 2023[170]). Furthermore, the primary m6A-binding molecules consist of YTHDF1/2/3 and YTH domain-containing proteins (YTHDC1 and YTHDC2). These proteins can precisely identify and attach to RNA molecules that have been altered with m6A (Lei and Wang, 2022[61]). m6A can regulate the activity of many genes associated with autophagy and promote the development of autophagosomes, as well as enhance the functioning of lysosomes (Hao et al., 2022[43]; Zhou, 2023[177]). 

Numerous investigations indicate that m6A may have a significant impact on regulating the process of neuron deterioration and influencing the progression of various neurological illnesses (Hao et al., 2023[42]; Peng et al., 2022[99]).

According to reports, m6A is found in high concentrations in the brain and has a significant impact on the growth of the CNS and the occurrence of disorders related, such as ischemia, ICH, PD, AD, and epilepsy (Lv et al., 2023[81]). Alterations in m6A modification within the CNS lead to aberrant neurological processes, such as impaired brain growth, synaptic breakdown, and alterations in cognitive and memory abilities (Lei and Wang, 2022[61]). YTHDF2 potentially controls the course of intracerebral hemorrhage (ICH) by influencing the ceRNA system and facilitating the invasion of M1 macrophages (Zhou, 2023[177]). After a period of reduced blood flow (ischemia), the reduction in the FTO enzyme may control the levels of the MEG3 gene in a way that depends on the m6A modification. This activation of neuronal pyroptosis occurs via the NLRP3/caspase-1/GSDMD cascade, ultimately resulting in impairment of the brain due to ischemia (Yan et al., 2024[153]). Within the context of PD, NRF1 can enhance the activity of glutaredoxin (GLRX) m6A, which in turn reduces motor impairment and the degradation of dopamine neurons. This effect is achieved by increasing the transcription of METTL3, as shown by Gong et al (Gong et al., 2024[40]). In AD, there is a potential decrease in the expression of METTL3 in the hippocampus of human AD samples. Conversely, increasing the expression of METTL3 might enhance the removal of phosphorylated Tau protein by autophagy and improve AD symptoms, as seen in laboratory experiments (*in vitro*) and animal models (*in vivo*) (Tang et al., 2023[126]). Furthermore, FTO also facilitates the development of AD by specifically affecting the TSC1-mTOR-Tau signaling pathway (Li et al., 2018[67]). The FTO enzyme may enhance the activity of the BDNF-TrkB network via m6A alteration, leading to the facilitation of reconsolidation of memory for recognizing new objects (Chang et al., 2023[15]). Nevertheless, the precise regulation process of m6A in epilepsy remains unclear, and abnormal m6A modulators may have a significant role in the initiation and progression of epilepsy.

Prior studies have shown that m6A alteration potentially serves in the regulation of synaptic breakdown, nerve injury, cognitive impairment, and neural growth anomalies in individuals with epilepsy. Currently, the m6A genes have been verified to exhibit variations between epilepsy sufferers and healthy persons using appropriate testing (Liu et al., 2022[76]). A recently published report presented a new form of FTO that does not include catalytic sites. This variation is associated with a distinct individual phenotype characterized by bilateral multifocal epilepsy and congenital abnormalities affecting many systems (Mayman et al., 2023[88]). Downregulation of FTO may lead to a rise in Nrf2 m6A alterations, which in turn promotes hippocampus cell damage and exacerbates the course of epilepsy in a rat model (Tian et al., 2023[131]). Additionally, EGR1 can trigger METTL3-associated suppression of VIM, which helps to reduce neuronal damage in epilepsy (Dong et al., 2023[31]). These data suggest that m6A alteration has a role in neuronal injury in epilepsy. The cerebellum of mice lacking the METTL3 gene is greatly reduced in size, resulting in severe atrophy. Additionally, the overall weight of the brain and cerebellum is dramatically decreased in these animals (Wang et al., 2018[135]). Deficiency in ALKBH5 may lead to disruption of the m6A alteration in genes associated with the formation of the cerebellum (Ma et al., 2018[82]). The data indicate a strong correlation between m6A and brain growth. 

Suppression of FTO in axons leads to an upsurge in m6A modification of Growth associated protein 43 (GAP-43) mRNA, resulting in a decrease in the translation of GAP-43 mRNA and the inhibition of axons (Yu et al., 2018[158]). Investigations have demonstrated that the absence of YTHDF1 or YTHDF3 results in synaptic disarray, as seen in a study by Merkurjev et al (Merkurjev et al., 2018[90]). Epileptogenesis in epilepsy is strongly linked to the synaptic excitatory receptor NMDAR. Gowda et al. (2022) showed that blocking FTO activity hinders the alteration of m6A levels induced by synaptic excitatory receptor NMDAR. Nevertheless, when FTO is upregulated in dopaminergic neurons (DNs), it decreases the amount of mRNA m6A, stimulates the production of NMDAR1, and encourages OS and Ca^2+^ influx, ultimately leading to the degradation or death of neurons (Li et al., 2018[67]). These investigations show that the disruption of the m6A alteration may hinder the development and functioning of synapses in epilepsy. The process of METTL3-mediated m6A mRNA alteration improves the capacity for learning and memory (Zhang et al., 2018[172]). The lack of YTHDF1 in the hippocampus of adult mice may result in impairments in memory and cognition. However, the reintroduction of YTHDF1 can restore the harm caused by this dearth (Shi et al., 2018[117]). YTHDF1, a reader of m6A, may enhance the CREB-BDNF cascade to enhance neuronal pyroptosis and cognitive impairment caused by sevoflurane (Huang et al., 2023[50]). According to Walters et al. (2017[134]), FTO is present in the CA1 area of mice's hippocampus, and the regulation of the development of memory is influenced by adverse feedback of FTO (Walters et al., 2017[134]). Furthermore, the METTL3 enzyme's involvement in m6A alteration enhanced the synthesis and development of pri-miR-221, resulting in an upregulation of miR-221-3p. This, in turn, exacerbated cognitive impairments in rats, as shown by Niu et al (Niu et al., 2022[96]). Those investigations indicate a correlation between m6A and the development of cognition and memory. In addition, VPA may also stimulate the production of FTO. Furthermore, when FTO is suppressed, it counteracts the detrimental impact of VPA on MBD2 and Na1.3 levels in epilepsy, as shown by Tan et al (Tan et al., 2017[123]). The results indicate a strong correlation between m6A and epilepsy. Nevertheless, the study on m6A in epilepsy remains restricted. 

Simply put, regulating m6A will help uncover the root pathophysiological process of neuropsychiatric disorders. The m6A alteration likely has a role in controlling synaptic disarray, nerve injury, cognitive impairment, and brain growth anomalies in individuals with epilepsy. Regulating m6A in the brain might be a promising approach for treating CNS disorders, such as epilepsy and associated cognition and memory deficits. This topic requires additional investigation in the field of human epilepsy.

## Clinical Implications of m6A Dysregulation in Epilepsy

### Potential of m6A as diagnosticbiomarkers 

m6A offers great potential as a diagnostic biomarker in epilepsy because of its regulatory function in gene expression and its consequences in numerous diseases, especially neurological diseases. Epilepsy can have several origins, and its diagnosis and treatment may depend on a clear knowledge of its molecular underpinnings. Known as m6A modifications, reversible chemical changes in RNA molecules significantly affect RNA stability, splicing, translation, and destruction (Deng et al., 2023[27]; Zhang et al., 2021[169]). Consequently, these changes from the main basis of control of gene expression. Particularly in the realm of cancer diagnostics, recent research has shown the possible value of m6A as a diagnostic biomarker (Bi et al., 2019[9]; Zhao et al., 2020[174]). Apart from its function in cancer, m6A has diagnostic importance for several diseases including epilepsy.

Research studies on the connection between m6A regulators and epilepsy point to their possible use as diagnostic biomarkers. Studies have found distinctly expressed m6A-related genes in both persons with and without epilepsy (Chen et al., 2024[17]; Liu et al., 2022[75]). Differential expression of seven major m6A regulator genes in epilepsy patients was found by analysis of the Gene Expression Omnibus (GEO) GSE143272 dataset. Among these genes are RBMX, HNRNPC, WTAP, RBM15, YTHDC1, YTHDC2, and CBLL1. Further confirming these results was the building of a random forest (RF) model, which underlined the relevance of these genes in estimating the risk of epilepsy. Decision curve analysis (DCA) was applied to construct a nomogram model to forecast epilepsy occurrence. Based on seven m6A regulators, this model has proven successful for epileptic patients (Chen et al., 2024[17]; Zhang et al., 2023[167]). The study divided epileptic patients into two distinct m6A patterns (cluster A and cluster B) using chosen significant m6A regulators to do consensus clustering. This enabled the identification of distinct immune cell elements and varying cell death patterns within each cluster. This classification emphasizes how complex m6A mutations interact with the immune response in epilepsy. Furthermore, the expression levels of important m6A-related genes were confirmed by quantitative real-time polymerase chain reaction (qRT-PCR) in blood samples taken from epilepsy sufferers, therefore confirming their differential expression (Chen et al., 2024[17]; Zhang et al., 2024[163]). For instance, whilst HNRNpc was expressed at a lower level in keeping with the findings of the integrated investigation, in patients with epilepsy WTAP and YTHDC1 displayed higher expression levels. This validation emphasizes the possible value of m6A regulators as epilepsy biomarkers. m6A's participation in vital cellular functions clarifies its epilepsy part even more. Especially in alternative splicing, cell cycle control, and the invasion of cancer cells, HNRNPC is important in pre-RNA processing. X-chromosome inactivation, cell cycle control, and alternative splicing are under WTAP's purview. By aggregating the m6A complex to specific RNA locations, RBM15 functions in several regulatory processes including the movement and proliferation of cancer cells (Chen et al., 2024[17]; Villa et al., 2019[133]). Both in the control of mitosis and meiosis and in mRNA degradation, the YTH domain-containing proteins which include YTHDC1 and YTHDC2 are quite important. RBMX and CBLL1 respectively are vital for the control of cell cycles and DNA damage repair. Including m6A-based precision medicine approaches in epilepsy could transform the diagnosis and treatment of this neurological condition. By using m6A regulators' prediction powers, doctors might create tailored treatment plans and improve diagnostic device accuracy. For instance, the m6A score obtained from important m6A regulators can be used to forecast pharmacodynamic reactions in epileptic patients, therefore directing drug choice and maximizing treatment outcomes. Moreover, the connection between glucose metabolism and m6A patterns in epilepsy emphasizes the more general consequences of m6A changes in metabolic processes related to the disease. This link could help novel therapeutic approaches aiming at metabolic pathways in epilepsy to be developed, thus improving the role of m6A as both a diagnostic biomarker and treatment target (Çarman et al., 2023[14]; Manna et al., 2022[84]; Villa et al., 2019[133]).

### Prognostic significance of m6A modifications in epilepsy outcomes 

Research on m6A alterations has attracted a lot of interest in connection to the development of epilepsy. m6A, the most frequent internal modification in eukaryotic mRNA, regulates translation, mRNA stability, and gene expression. m6A-associated proteins-comprising methyltransferases (writers), demethylases (erasers), and m6A-binding proteins (readers)-have been linked to various diseases, including epilepsy (Wang et al., 2024[137]). Comparatively to those without epilepsy, a recent study using GEO GSE143 has found clearly expressed m6A regulators in those with the disorder. The following genes show notable changes: RBM15, YTHDC1, CBLL1, RBMX, WTAP, and HNRNPC (Zhang et al., 2022[166]). Crucially important physiological processes including alternative splicing, cell cycle control, and mRNA stability depend on these genes. These m6A regulators helped to build a predictive model to evaluate therapy efficacy and epilepsy susceptibility. This model showed possible clinical value and proved able to guide therapy decisions and project patient outcomes. Moreover, different m6A alteration patterns were found that matched genes linked to autophagy, ferroptosis, and cuproptosis (Tang and Lv, 2021[125]; Wang et al., 2024[137]). This suggests that channels of cell death in epilepsy may be influenced by m6A modifications. The findings suggest that m6A patterns might be biomarkers for distinct forms of epilepsy and help to guide treatment approaches. 

m6A changes are indicative of several diseases, including acute myeloid leukemia (AML), and outside epilepsy. Under AML, a prediction model based on m6A alterations (m6APR_Score) showed notable accuracy in estimating patient survival over several datasets. This emphasizes m6A changes' strong resilience as prognostic indicators (Zhang et al., 2020[165]). By analyzing 17 m6A-related genes from a publicly available dataset, researchers explored the significance of m6A modifications in predicting epilepsy outcomes. Among these, seven genes-WTAP, HNRNPC, RBM15, CBLL1, YTHDC1, YTHDC2, and RBMX-exhibited distinct expression patterns between healthy individuals and those with epilepsy. Based on their relative relevance, an RF model ranked these genes; a nomogram was developed from these genes to forecast epileptic incidence. This model shows therapeutic advantages via DCA curve analysis. Still, the small number of genes tested hampered the research. Furthermore, linked to the immune reaction in epilepsy are m6A regulators (Lin et al., 2022[71]; Wu et al., 2021[150]). The distinct expression of m6A regulators strongly correlates with the presence of specific immune cell components, offering a basis for exploring the connection between m6A and autoimmune epilepsy. Autoantibodies targeting leucine-rich glioma-inactivated protein (LGI1) and glutamic acid decarboxylase (GAD) play critical roles in autoimmune epilepsy. Examining m6A and cell death pathways-cuproptosis, pyroptosis, and ferroptosis-has finally shown a significant link between iron death and epilepsy. This link emphasizes the growing possibility of m6A changes as indicators for comprehending the complicated processes behind epilepsy and thereby enhancing patient outcomes (Zhao et al., 2024[173]). 

### Therapeutic targeting of m6A machinery in epilepsy management

Targeting the m6A machinery presents interesting therapeutic approaches for the control of epilepsy. With an eye toward LIM kinase 1 (LIMK1), MiRNA-134 is elevated in TLE particularly concerning epilepsy (Tian et al., 2023[131]; Wang et al., 2024[137]). Epilepsy develops in part from the increase in LIMK1 translation decrease. Recently a study found a possible m6A site in miRNA-134 and reasoned from this that m6A methylation influences its binding capacity to target mRNAs. Since miRNA-134 has shown neuroprotective and antiepileptic benefits, targeting its m6A methylation will help to efficiently control its negative effects in epilepsy. By synthesizing a chemical known as 7d, which functions as an inhibitor for the demethylase FTO, Zheng has devised a novel pharmaceutical approach (Shen et al., 2021[114]; Tian et al., 2023[131]). Compound 7d reduces FTO therefore improving m6A methylation levels in cells and has anticonvulsant effects in mice. This suggests that to lower epileptic activity, FTO inhibitors could be able to control m6A methylation. Although the exact mechanisms by which medication 7d stops seizures are yet unknown, this finding emphasizes the possibility of reducing FTO in the treatment of epilepsy. Differential expression patterns in epileptic patients have been found through m6A-related gene analysis. RNA processing, cell cycle control, and mRNA decay all depend on these genes in great part. YTHDC1 and YTHDC2 among other YTH family members interact with m6A-modified RNAs to control mRNA splicing and degradation respectively (Lv et al., 2023[81]; Tian et al., 2023[131]). 

Targeting these genes linked to m6A alteration and their related pathways could provide fresh approaches for controlling epilepsy. Whereas changes in m6A have been linked with changes in immune cell components, epilepsy has been linked with immune system dysregulation. The hallmark of autoimmune epilepsy is the development of autoantibodies aiming at neuronal surface targets. The direct effects of this disorder are on synaptic activation and plasticity. The variable expression of m6A in epilepsy can affect immune responses; so, interventions aiming at m6A changes have a basis in terms of regulating immune-mediated epileptic processes. Important pathogenic traits of epilepsy are neuronal loss and degeneration (Zhang et al., 2022[171]). Research on m6A has revealed that changes in this molecule affect several types of programmed cell death (PCD), including ferroptosis, closely linked to epilepsy (Huang et al., 2022[49]; Liu et al., 2022[76]). Directing focus on m6A methylation in pathways linked to neuronal death helps to minimize these aberrant changes and maybe lowers the frequency and intensity of seizures (Chen et al., 2024[17]). 

Studies on m6A metabolism's related processes-the tricarboxylic acid (TCA) cycle and pyruvate metabolism-show how likely epilepsy could develop from them. Therapies aiming at m6A changes in metabolic pathways could have two advantages in treating metabolic diseases as well as epilepsy (Lv et al., 2023[81]; Tian et al., 2023[131]). Using therapeutic approaches to target the m6A machinery in epilepsy management presents a different way of treatment. Targeting genes expressed differently concerning m6A, controlling m6A methylation using inhibitors such as compound 7d, and suppressing damaging miRNAs like miRNA-134 constitute effective techniques Furthermore creating thorough therapeutic options depends on an awareness of the interactions among m6A alterations, immunological responses, neuronal cell death, and metabolic pathways (Shen et al., 2021[114]; Suga et al., 2023[120]; Tian et al., 2023[131]).

## Therapeutic Strategies Targeting m6A in Epilepsy

### Modulation of m6A writers, erasers, and readers 

m6A in RNA is changed under dynamic interaction among enzymes known as "writers," "erasers," and "readers." Each of these enzymes adds, removes, and interprets the m6A alteration in turn: From splicing to export, stability to translation, these enzymes affect many facets of RNA metabolism. They so can affect several physiological processes and disease paths. An MTC mostly serves to help m6A be installed. Among these intricate elements are METTL3, METTL14, WTAP, KIAA1429, RBM15, and ZC3H13. METTL3's catalytic core shows a predilection for methylating areas in RNA including the sequences GAC and AAC (Dai et al., 2021[25]; Fang et al., 2022[34]). Localization to nuclear speckles suggests a function in mRNA splicing and other nuclear processes. For embryonic development, cell reprogramming, and spermatogenesis, METTL3 is essential. Early embryonic death in mice results from METTL3 deletion. Using specific target gene methylation, METTL3 controls T cell homeostasis and endothelial-to-hemopoietic transition. Crucially important to the MAC, METTL14 forms a stable heterodimer with METTL3 (Shi et al., 2019[116]). While METTL14 mostly serves as an RNA-binding scaffold to improve the catalytic efficiency of METTL3, METTL3 shows enzymatic activity. From embryonic stem cells' self-renewal to differentiation and neurogenesis, the METTL3-METTL14 complex has been linked to several biological activities. For instance, lack of METTL14 results in less-than-ideal gametogenesis and developmental abnormalities. Fascinatingly, METTL14 both acts as an inhibitor of tumor development in glioblastoma and helps cancer cells proliferate in AML (Feng et al., 2023[36]; Wang et al., 2020[139]). Using METTL3 and METTL14, WTAP controls m6A levels in RNA transcripts. Although WTAP lacks catalytic activity, it is crucial for anchoring the METTL3-METTL14 complex within nuclear speckles (NS). Like the problems seen in zebrafish embryos lacking METTL3, the depletion of WTAP causes variations in tissue-specific abnormalities. Although the precise function of WTAP in m6A control in AML is yet unknown, it is certain that in more than 30 % of AML cases, WTAP is greatly elevated, suggesting a possible role in cancer development promotion. Comprising a part of the MAC, KIAA14 significantly influences m6A methylation. For example, m6A levels significantly drop when KIAA14 is deleted from human cells. Emphasizing its relevance in the complex's regulation, KIAA1429 is mostly responsible for guiding region-specific m6A methylation. Directing methylation of adenosine residues in both mRNAs and the lncRNA XIS is the work of RBM15 and its paralogues (Chen et al., 2020[18]; Zhou et al., 2020[178]). The relevant proteins can attach to the WTAP-METTL3 complex and draw it to RNA locations. They therefore help to guide the installation of m6A only at those exact places. Drosophila provides more evidence of the function of the RBM15 homolog Spenito in the MTC since m6A in mRNAs is produced depending on it. ZC3H13, a recently identified component of the m6A writer complex, is crucial for enabling the nuclear localization of the MTC. It plays a vital role in various processes, including sex determination in *Drosophila* (Flamand et al., 2023[39]; Meyer and Jaffrey, 2014[91]). It combines with RBM15 and WTAP to help m6A be added to mRNAs. The enzymes ALKBH5 and FTO mediate the removal of m6A by catalyzing its demethylation. FTO proved to be the first enzyme showing rather high m6A demethylase efficiency. Oxidation of m6A generates intermediaries including N6-hydroxymethyladenosine and N6-formyladenosine. Strongly abundant in the brain, FTO is linked to obesity. Apart from its role as an oncogene in glioblastoma and leukemia, it accelerates leukemia development by lowering the m6A levels in important transcripts. Another demethylase, ALKBH5, is abundantly produced in the testes and is important for several physiological processes. By allowing the dynamic regulation of m6A, it influences RNA RNA and several metabolic activities. Proteins containing YTH domains contribute to RNA metabolism by recognizing and binding to m6A-modified RNA. By decoding the m6A tags, these proteins help to enable actions including mRNA degradation and translation. YTHDF proteins improve the stability and efficiency of mRNA translation, therefore influencing gene expression following transcription (Tegowski and Meyer, 2024[129]).

### RNA modification editing technologies 

RNA modification editing technologies are emerging as promising tools in the development of novel treatments for epilepsy. These advanced techniques allow for precise alterations of RNA molecules, potentially modulating gene expression and protein function in epileptic brain tissue. One key approach involves adenosine-to-inosine (A-to-I) editing, mediated by adenosine deaminases acting on RNA (ADARs). This method can be used to target specific RNA transcripts involved in neuronal excitability and synaptic transmission, potentially reducing seizure frequency and severity (Qiu et al., 2023[107]; Yang et al., 2021[154]). Another promising strategy utilizes CRISPR-Cas13 systems, which can be programmed to target and modify specific RNA sequences with high precision. This technology offers the potential to correct disease-causing mutations or alter the expression of genes implicated in epileptogenesis (Cox et al., 2017[23]). Additionally, m6A modification, the most prevalent internal modification in eukaryotic mRNA, has been shown to play a role in neuronal function and could be manipulated to modulate epileptic activity. Researchers are also exploring the use of antisense oligonucleotides (ASOs) to induce RNA editing, potentially correcting splicing defects or modifying protein-coding sequences relevant to epilepsy (Morris et al., 2021[94]). While these RNA modification editing technologies show great promise, challenges persist regarding off-target effects, delivery methods, and long-term safety (Asmamaw and Zawdie, 2021[6]; Birgaoanu et al., 2023[10]; Shinwari et al., 2018[119]). Current research is centered on enhancing the specificity and efficiency of these techniques, as well as developing targeted delivery systems to ensure their effects are limited to epileptic brain regions. As our knowledge of the molecular mechanisms behind epilepsy continues to grow, RNA modification editing technologies may provide a powerful means of developing personalized, precision treatments for this complex neurological disorder.

All symptoms of epilepsy have a shared hallmark of aberrant hyperactivity in a specific group of neurons, resulting in significantly increased amounts of intracellular calcium ions. Neuronal cells subjected to sustained and non-lethal elevations in Ca^2+^ ion levels experience plastic alterations, leading to the development of epileptogenesis. AMPA glutamate receptors are crucial in facilitating rapid excitatory neurotransmission and are believed to serve some involvement in the development of convulsions. Previous investigations conducted on mouse models have shown that a deficiency in GluA2 Q/R RNA editing is associated with an increased susceptibility to seizures (Brusa et al., 1995[12]; Higuchi et al., 2000[45]). This idea is supported by the research, which found a clear correlation between the extent of disruption of GluA2 Q/R RNA editing and the level of Ca^2+^ leakage (Feldmeyer et al., 1999[35]). Additionally, mice with a specific genetic mutation that prevented GluA2 editing in some parts of the brain after birth exhibited synaptic alterations that suggested an increased vulnerability to experiencing seizures (Krestel et al., 2004[59]). The information presented indicates that the epileptic characteristics seen in GluA2 Q/R editing-deficient mice models may be directly caused by changes in AMPA-receptor characteristics in adult brains, rather than being an outcome of developmental abnormalities.

The precise biochemical process behind the development of epilepsy resulting from the impairment of GluA2 Q/R site alteration remains unknown. Despite the increased permeability of AMPA receptors missing GluA2 subunits to Ca^2+^ ions, GluA2 silencing animals have normal survival and do not display any seizure-related characteristics (Jia et al., 1996[53]). This difference may be explained by the influence of the Q/R position in the GluA2 component, which influences not only the capacity of Ca2+ to pass through, but also the speed at which the channel opens and closes, the flow of ions through the channel, the formation of the channel, and the movement of the channel within the cell. While there is ample proof supporting the causal link between GluA2 Q/R RNA editing and epilepsy in mice models, the findings from investigations on GluA2 Q/R editing in clinical samples from individuals with epilepsy are inconclusive. A research investigation conducted on needle biopsy specimens taken from hypothalamic hamartoma cells, which is believed to be an inherent component of epilepsy, discovered a reduction in nuclear immunostaining of ADAR2 together with a decline in RNA editing effectiveness at the GluA2 Q/R site (Kitaura et al., 2017[55]). Nevertheless, several investigations have shown that the reduction of GluA2 Q/R site editing was not detected in the surgically removed hippocampus and temporal cortex tissues of individuals with epilepsy (Kortenbruck et al., 2001[57]; Krestel et al., 2013[58]). Additional research is required to fully comprehend the pathogenic significance of GluA2 Q/R site modification in epilepsy.

### Challenges and opportunities in developing m6A-targeted therapeutics

Treatments meant especially to target m6A for epilepsy present both great potential and great difficulties. Regulation of gene expression is greatly aided by the RNA modification m6A. Particularly in situations refractory to present treatments, knowledge of the capacity to control m6A circuits could offer fresh approaches for treating epilepsy. Developing m6A-targeted treatments for epilepsy is much challenged by the intricacy of the m6A alteration process itself. m6A is enzymatically added to RNA by METT; demethylases can thus undo its presence. Moreover, binding proteins can find and mark m6A changes. The complex interactions among them make it challenging to identify certain therapeutic intervention targets (Deng et al., 2022[26]; Mao et al., 2024[85]). The main m6A METT is the METTL3-METTL14 complex; FTO and ALKBH5 function as demethylases. Any therapeutic approach must consider the balance between these conflicting forces if one is to properly control m6A levels without interfering with normal cellular activities. Giving m6A-targeted treatments to the brain is still another difficult task. Most medications find the blood-brain barrier (BBB) to be a major obstacle; adding RNA-based therapies makes matters more difficult. Effective ASOs and other medications targeting RNA must pass the BBB and reach the neurons in the right concentrations (Jo et al., 2023[54]). Although direct intracerebral injections are now the most efficient way to administer these treatments, this approach is invasive and unworkable for general clinical use. Furthermore, needed are strong and consistent markers to track the effectiveness of m6A-targeted therapy for epilepsy. Targeting treatments depends on the discovery of particular m6A alterations linked to epileptogenesis and seizure activity. Still, the often shifting and context-specific character of m6A changes presents a difficulty. Biomarkers must be sufficiently sensitive to find variations in m6A levels and sufficiently specific to separate pathogenic changes from normal variations in RNA methylation (Liu et al., 2022[74]). Notwithstanding these difficulties, m6A-targeted therapies in epilepsy have great chances for development. The possibility of m6A alterations presents an interesting path to offer a new class of disease-modifying drugs. Unlike present antiseizure medications that mostly treat symptoms, m6A-targeted therapy can change the fundamental disease processes. By changing the expression of genes linked to neuronal excitability and synaptic function, drugs targeted at m6A, for instance, could lower the frequency and intensity of seizures or possibly stop the start of epilepsy in people at risk. Moreover, a major benefit of RNA-based treatments including ASOs is their modifiability or adjustability (Zhang et al., 2022[166]). ASOs permit exact control of m6A levels on some transcripts by especially targeting RNA sequences. This accuracy could be crucial in creating medicines with great concentration as their few off-target effects suggest their simplicity (Tegowski and Meyer, 2024[129]). 

Recent improvements in ASO chemical modification have enhanced their stability and potency, hence increasing their therapeutic value. Moreover, the growing knowledge of the m6A epitranscriptome in many diseases presents the chance for tailored treatment to treat epilepsy. Individual patient profiles of the m6A landscape could help to enable tailoring of therapy to their epigenetic changes. Using this tailored strategy might help to improve therapy results and lower the risk of side effects. For a patient with drug-resistant epilepsy, for example, particular treatments could be created to especially undo an m6A alteration seen to be increased. Already showing promise in tackling m6A circuits in neurological diseases is preclinical research. Improving cognitive function has been demonstrated by blocking the m6A reader protein YTHDF1 in animal models of AD. In models of epilepsy, different strategies could be investigated to evaluate their effects on the course of the condition and frequency of seizures. These findings might open the path for clinical trials and final clearance of m6A-targeted epilepsy treatments (Feng et al., 2023[36]). 

## Future Directions and Emerging Research Frontiers

The current explosion of research has concentrated on revealing fresh targets and mechanisms linked with m6A changes, therefore providing a fresh understanding of their functional relevance and possible therapeutic uses. The discovery of new m6A targets has been facilitated by advancements in high-throughput sequencing technologies and bioinformatics approaches. Techniques such as m6A-seq and miCLIP (methylation individual-nucleotide resolution crosslinking and immunoprecipitation) have enabled the exact identification of m6A sites across the transcriptome achievable. Several m6A-modified sites have been found by these technologies, therefore exposing the broad and dynamic character of this change (Villa et al., 2019[133]). Several RNA molecules, including mRNA, lncRNA, miRNA, and rRNA, have shown the presence and regulatory influence of m6A alterations, so stressing their general occurrence. According to recent research, several biological activities are much influenced by m6A changes. Stability, splicing, export, translation, and destruction are only a few of the several facets of RNA affected by changes in mRNA m6A. Specific reader proteins, such as YTHDF1, YTHDF2, and YTHDF3, are recruited to recognize and bind to m6A-modified RNA, thereby improving mRNA stability and enabling translation (Zhou et al., 2020[178]). These reader proteins help to either recruit translation initiation factors or encourage the breakdown of mRNA, therefore controlling post-transcriptional gene expression. 

One of the main instances of m6A's functional relevance is its control of stem cell proliferation and differentiation. Changing important transcription factors and signaling molecules has revealed that m6A changes can affect embryonic stem cell destiny. Control of the transcription factor NANOG via m6A alteration significantly affects the preservation of the capacity of embryonic stem cells to develop into distinct types of cells. Similar m6A changes have been linked to the differentiation of NSC, where they control the expression of genes involved in neurogenesis and neuronal development (Liu et al., 2022[74]). One important line of study is looking at how m6A mutations could cause cancer. Leukaemia, breast cancer, and liver cancer are among the several forms of cancer linked to aberrant m6A modification patterns. Studies have revealed, for example, that changes in m6A play a critical role in controlling the capacity of leukemia stem cells to self-renew and differentiate, therefore influencing the course of the disease. Targeting the m6A machinery-including FTO, a demethylase-or METTL3-has become a possible approach for cancer treatment. Targeting m6A alterations in cancer has therapeutic potential since METTL3 inhibitors have been demonstrated to stop leukemia cell proliferation and improve their sensitivity to chemotherapeutic therapies (Zhang et al., 2020[165]; Zhang et al., 2022[166]). In the field of neurology, m6A alterations show promise, especially concerning epilepsy. Recurrent convulsions define epilepsy, which typically shows resistance to traditional therapies. Recent studies show that m6A changes may shed light on the basic causes of epilepsy by modulating the activity of genes linked to the excitability of neurons and synapse functioning. Dysregulation of m6A methylation in animal models of epilepsy suggests that focussing treatment on m6A pathways could provide fresh therapeutic opportunities. More specifically, changing m6A levels in brain circuits might assist in controlling neuronal activity and lower seizure frequency (Cox et al., 2017[23]; Li et al., 2022[66]).

New pathways linked to m6A mutations have improved our knowledge of their regulating functions. m6A changes in circular RNAs (circRNAs), a form of ncRNA with regulating roles, have been shown recently. Evidence shows that changes in m6A within circRNAs influence their stability and translation, so influencing gene expression (Shen et al., 2021[114]). Furthermore, linked to the management of RNA-protein interactions and the development of RNA-protein complexes, which are fundamental for many biological processes, are m6A alterations. The identification of m6A "writers," "erasers," and "readers," highlights even more the dynamic and reversible character of m6A changes. Whereas METTL3 and METTL14 are writers that catalyze the addition of m6A marks to RNA, FTO, and ALKBH5 are erasers that remove m6A marks from RNA. Readers such as YTH domain-containing proteins can find and bind to RNA molecules altered with m6A. This helps them control the later consequences of the alteration. The interactions among these proteins guarantee exact control of m6A alterations and their functional effects (Monian et al., 2022[93]).

### Harnessing multi-omics approaches to study m6A in epilepsy 

Using multi-omics techniques to investigate m6A in epilepsy marks a future direction in knowledge of the intricate processes behind this neurological condition. Particularly concerning RNA modifications like m6A, the combination of multi-omics technologies-genomics, transcriptomics, epigenomics, and proteomics-offers a whole picture of the molecular terrain of epilepsy. Often with a complicated etiology comprising genetic, epigenetic, and environmental elements, epilepsy is defined by repeated spontaneous seizures. Although it targets ion channels and neurotransmitter systems most of the time, traditional pharmacotherapy is useless for roughly thirty percent of patients. This has piqued curiosity about other therapeutic targets, including RNA alterations (Deng et al., 2022[26]; Zhang et al., 2022[171]). m6A, the most prevalent internal modification in eukaryotic mRNA, plays a crucial role in various biological processes, including splicing, translation, localization, and mRNA stability. Advances in bioinformatics tools and high-throughput sequencing methods recently have greatly improved our capacity to map m6A changes over the transcriptome. High-resolution m6A site identification has been much aided by methods such as m6A-seq, which combines immunoprecipitation of m6A-modified RNA with next-generation sequencing. These methods let scientists investigate the dynamic variations in m6A methylation in response to various physiological and pathological states, including epilepsy (Liu et al., 2022[75]; Wu et al., 2021[150]). In the framework of epilepsy, for example, research has revealed that m6A alterations are actively controlled during epileptogenesis. Crucially m6A METTL3 has been linked to control of neuronal survival and function. Changing the levels of m6A-related enzymes including METTL3 has shown in experimental models of epilepsy that this influences seizure susceptibility and neuronal excitability. In one study, a common precursor to epilepsy, traumatic brain injury, caused notable changes in m6A-modified mRNA transcripts in the hippocampal suggesting possible therapeutic targets for early intervention. Multi-omics techniques let different layers of molecular information be merged with these epitranscriptomic data. Combining m6A-seq with RNA-seq (transcriptomics) for instance can show how m6A alterations affect gene expression profiles in epileptic tissues (Tegowski and Meyer, 2024[129]). Including proteomics data can also help to clarify how m6A influences protein synthesis and function. Utilizing an all-encompassing approach, important regulatory networks and pathways disturbed in epilepsy can be found, therefore offering information on possible therapeutic targets. Cell-type specificity of RNA alterations presents one of the main difficulties in researching m6A in epilepsy. The brain is a diverse organ with different cell types displaying different molecular fingerprints. Combining m6A mapping methods with single-cell RNA sequencing (scRNA-seq) offers cell-type-specific insights on m6A dynamics, so addressing this difficulty. This method can reveal how distinct neuronal and glial cell populations contribute to the pathophysiology of epilepsy and how m6A alterations modify their function (Feng et al., 2023[36]). 

Furthermore, combining m6A profiling with epigenomic data-like histone modification and DNA methylation-allows one to have a more complete knowledge of the regulating processes underlying epilepsy. Epigenetic changes can affect m6A deposition on RNA, and vice versa, therefore producing a complicated interaction controlling gene expression and neural activity. Knowing these relationships helps one to find fresh epigenetic and epitranscriptomic targets for treatment. Regarding therapeutic development, focusing on m6A alterations offers both possibilities and difficulties. The reversible character of m6A alterations presents a possible therapeutic intervention path. Small compounds or ASOs meant to control m6A-related enzyme activity could restore normal m6A levels and help to reduce illness symptoms. In preclinical animals, for instance, suppression of miRNAs using ASOs has shown promise in lowering seizure frequency and severity (Villa et al., 2019[133]; Zhou et al., 2020[178]).

### Integration of m6A-based precision medicine approaches

Application of m6A-based precision medicine techniques to epilepsy shows the possibility for improved patient outcomes and tailored treatment. Typical targets of traditional antiepileptic drugs are neurotransmitter receptors and ion channels. On the other hand, there is a growing need for tailored treatments that consider every person's genetic and molecular makeup. One often occurring internal alteration in eukaryotic mRNA is m6A (Zhang et al., 2024[163]). Gene expression regulation is crucial for governing other vital processes including cellular self-renewal, differentiation, and mortality. Researchers are focusing on the reversible character of m6A alteration to help acquire a more complete knowledge of the etiology of many disorders. It involves writers (like METTL14 and METTL3), erasers (like ALKBH5 and FTO), and readers (like YTHDF2 and YTHDF1) among enzymes. m6A patterns may affect both the prognosis and the efficacy of treatment. Seven notable m6A regulators were found to be present in epileptic individuals, according to an examination of the GEO GSE143272 dataset. These regulators were successfully recognized by the RF model as reliable indicators of the risk of epilepsy (Booth et al., 2023[11]). These m6A regulators have been used in a nomogram model that has demonstrated encouraging practical application, suggesting that patients may benefit from individualized treatment plans based on their m6A profiles. Using the consensus clustering method, patients were categorized into two distinct m6A modification patterns, designated as cluster A and cluster B. This division revealed distinct variations in the makeup of immune cells and how cells die. Individuals in cluster B, who had higher m6A scores, showed different immune responses and metabolic profiles than those in cluster A (Bi et al., 2019[9]; Wang et al., 2024[137]). This differentiation is critical because it suggests that m6A mutations may affect immune system functions and metabolic pathways in epilepsy. This lays the groundwork for the application of precision medical techniques. Moreover, m6A patterns and glucose metabolism were found to be correlated an important finding in the treatment of epilepsy. Understanding these links between metabolisms can help with developing therapeutic strategies that target metabolic pathways in particular (Levanon et al., 2005[63]; Zhang et al., 2021[169]). This may result in the development of more effective and customized epilepsy medications. This approach aligns with the overall goals of precision medicine, which aim to tailor treatments based on unique genetic and molecular traits. The accomplishment of several crucial steps is necessary for the application of m6A-based precision medicine in epilepsy. To identify m6A mutations and the associated regulatory factors in each patient, comprehensive genomic and transcriptome investigations are first required (Chen et al., 2020[18]). 

Understanding the functional significance of m6A landscapes in epilepsy and correctly mapping them requires the application of bioinformatics methods and cutting-edge sequencing technologies. These studies have the potential to identify m6A patterns unique to each patient, which may then be connected to treatment outcomes and clinical manifestations. Research has revealed that m6A mutations can influence the expression of genes associated with key processes in epilepsy development, including autophagy, ferroptosis, and cuproptosis (Booth et al., 2023[11]; Zhang et al., 2024[163]). Innovative therapeutic approaches to control cell death and improve neuronal survival in epileptics can be developed by concentrating on these pathways. Furthermore, a thorough understanding of the molecular processes causing epilepsy can be acquired by combining m6A analysis with other omics data, such as proteomics and metabolomics, which will improve the accuracy of treatment techniques (Shi et al., 2023[118]). Creating clinical models to forecast treatment outcomes is essential to implementing m6A-based precision medicine. Machine learning algorithms, like support vector and RF models, have been used in recent studies to evaluate complex datasets and find treatment targets and predictive biomarkers. With consideration for each patient's unique m6A profile and clinical features, these models can help physicians decide on the best course of action (Fang et al., 2022[34]).

## Conclusion

Despite decades of research, epilepsy continues to evade complete control and burdens millions worldwide, challenging the boundaries of current therapeutics. This review underscores the critical importance of m6A RNA modification in epilepsy and the need for further investigation into how these modifications affect neuronal functions and disease development by regulating oxidative stress, synaptic plasticity, and mitochondrial dysfunction-key processes in epilepsy pathogenesis. Precision editing technologies and small-molecule interventions targeting m6A machinery offer promising therapeutic opportunities, particularly in drug-resistant epilepsy. Additionally, the use of m6A alterations as biomarkers may enable the development of personalized treatment strategies aimed at optimizing outcomes for individual patients.

However, while m6A-based interventions hold potential, several limitations must be acknowledged. The global and dynamic nature of m6A modifications raises concerns about specificity, as targeting core components of the m6A machinery (e.g., METTL3, FTO) may produce widespread transcriptomic effects, leading to unintended consequences. Moreover, the context-dependent roles of m6A-varying by brain region, developmental stage, and seizure type-complicate the identification of universal targets. The lack of selective modulators and effective delivery systems further hampers translational progress. Finally, our understanding of how m6A modulation intersects with other epigenetic and cellular pathways in epilepsy remains incomplete.

In summary, while the integration of m6A-based strategies into epilepsy care represents a paradigm shift, it must be approached with caution and guided by rigorous mechanistic studies. Continued research into the nuanced regulation and function of m6A methylation will be essential to unlocking its full therapeutic potential and delivering meaningful clinical advances for patients with epilepsy.

## Declaration

### Acknowledgements

The authors would like to thank the Deanship of Scientific Research at Tabuk University for supporting this work.

### Author Contributions 

Mudasir Maqbool: Writing - original draft, Data curation, Investigation. Md Sadique Hussain: Conceptualization, Investigation, Writing - original draft. Mohammed M. Arab: Methodology, Writing - original draft. Saud O. Alshammari: Data Curation, Software, Writing - Review & Editing. Yumna Khan: Formal Analysis, Investigation, Visualization. Fawaz M. Almufarriji: Conceptualization, Writing - Review & Editing.

### Ethics Approval

This review article was not subject to any ethics approval from the institutions.

### Informed Consent 

Not Applicable.

### Funding 

The authors declare that no funds, grants, or other support were received during the preparation of this manuscript.

### Declaration of competing interest 

The authors declare that they have no known competing financial interests or personal relationships that could have appeared to influence the work reported in this paper. 

### Data availability 

No data was used for the research described in the article.

### Use of Artificial Intelligence (AI)

During the preparation of this manuscript, the authors used ChatGPT, an AI-assisted language model, to correct grammatical and typographical errors. No content or image was generated by AI without human review. All authors have reviewed and approved the final version of the manuscript.

## Figures and Tables

**Figure 1:**
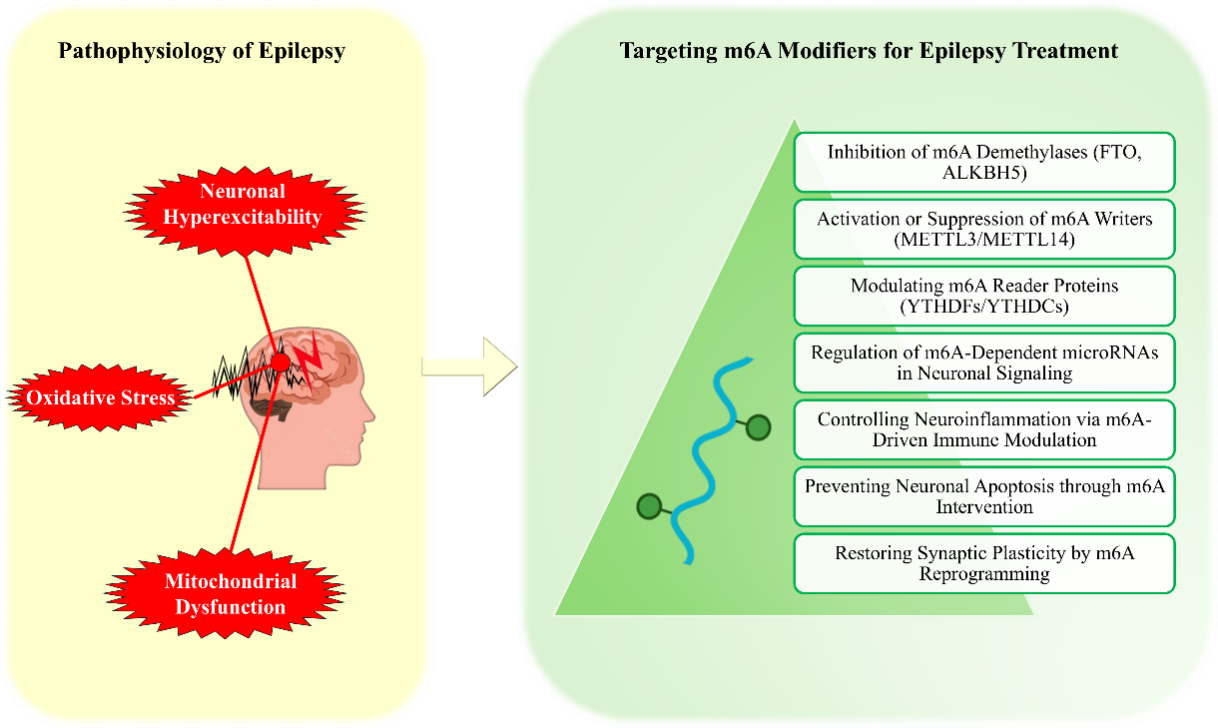
Graphical abstract
